# Nanostructured Lipid Carrier-Filled Hydrogel Beads for the Delivery of Curcumin: Digestion, Intestinal Permeation, and Antioxidant Bioactivity After Gastrointestinal Digestion

**DOI:** 10.3390/pharmaceutics17050541

**Published:** 2025-04-22

**Authors:** Rui Sun, Chenyu Wei, Xiaoyan Tang, Yufeng Sun, Juling Ji

**Affiliations:** Department of Pathology, Medical School of Nantong University, Nantong 226001, China; sunrui@ntu.edu.cn (R.S.); 2331310037@stmail.ntu.edu.cn (C.W.); 2213330010@stmail.ntu.edu.cn (X.T.); syf0817@ntu.edu.cn (Y.S.)

**Keywords:** nanostructured lipid carriers, hydrogel beads, in vitro digestion, ex vivo permeation, antioxidant bioactivity

## Abstract

**Background/Objectives:** The aim of the present study was to develop nanostructured lipid carrier (NLC)-filled hydrogel beads for the delivery of curcumin in functional foods. **Methods:** Curcumin-loaded NLC-filled hydrogel beads based on calcium alginate were developed using the extrusion method. Various preparation parameters, physicochemical characteristics, gastrointestinal fates, and antioxidant bioactivities were studied to confirm the feasibility of this delivery system. **Results:** Curcumin-loaded NLCs were successfully filled into hydrogel beads with an encapsulation efficiency above 80%. The stability test displayed that the stability of curcumin encapsulated within NLCs was further enhanced when the NLCs were filled into beads. During in vitro digestion, the lipolysis rate of the lipid matrix and the release rate of curcumin encapsulated in NLCs were adjusted by the hydrogel beads. The ex vivo intestinal permeation study indicated that the intestinal permeation of curcumin from the digestion products of curcumin-loaded NLC-hydrogel beads, prepared with appropriate alginate concentrations (0.5% and 1%), was significantly enhanced compared to that of curcumin-loaded NLCs. Furthermore, the digestion products of curcumin-loaded NLC-hydrogel beads (1% alginate) exhibited significantly enhanced antioxidant bioactivity compared to those of curcumin-loaded NLCs. **Conclusions:** This study demonstrated that NLC-hydrogel beads might be a promising delivery system for hydrophobic bioactive compounds in functional food systems.

## 1. Introduction

Curcumin, a low-molecular-weight hydrophobic polyphenol, is mainly isolated from turmeric [1]. Due to excellent biocompatibility and low toxicity, curcumin has been widely used in the food industry as a spice and dye. Recent studies have revealed that curcumin exhibits potential health benefits such as antioxidant, antitumor, antimicrobial, anti-inflammatory, hepatoprotective, antidiabetic, and anti-aging bioactivities [2]. However, its application in functional foods is restricted by its low water solubility, high melting point, and chemical instability, including pH sensitivity and photosensitization. In addition, due to the poor aqueous solubility and the degradation in the intestinal environment, the oral absorption and bioavailability of curcumin exhibit significant limitations [1]. Therefore, the fate of curcumin in the gastrointestinal tract requires special attention. In recent years, several lipid-based delivery systems, such as Pickering emulsions, nanoemulsions, double emulsions, lipid nanocapsules, lipid nanoparticles, and liposomes, have garnered significant attention from the food industry to overcome limitations in the application of curcumin [3,4,5,6,7,8].

Lipid nanoparticles are emerging delivery systems for hydrophobic bioactive compounds, offering advantages such as high biocompatibility, low toxicity, organic solvent-free processing, and high encapsulation efficiency. The first-generation lipid nanoparticles, termed solid lipid nanoparticles (SLNs), were developed by replacing liquid lipids in traditional oil-in-water nanoemulsions with solid lipids, forming a solid-state nanocarrier at room temperature [9]. Nanostructured lipid carriers (NLCs), considered the second generation of lipid nanoparticles, were developed by utilizing a blend of solid and liquid lipids as the lipid matrix [10]. Compared to SLNs, NLCs are considered to possess more lattice defects, which can be utilized to encapsulate hydrophobic bioactive compounds, thereby enhancing the encapsulation efficiency and stability. NLCs are commonly formulated using cost-effective and safe lipids, such as triglycerides, monoglycerides, and fatty acids, through scalable production methods including high-pressure homogenization, which provide them with significant industrial potential [10]. NLCs have distinct advantages over nanoemulsions while retaining some benefits of nanoemulsions. This solid matrix significantly restricts the mobility of encapsulated hydrophobic bioactive compounds, reducing the diffusion rate to the particle surface and minimizing interfacial reactions, such as oxidation [11]. However, the stability and gastrointestinal fate of NLCs restrict food industrial application. Although liquid lipids are added to the lipid matrix to improve storage stability, polymorphic transition during storage might still occur, leading to an increase in lipid exposure and the aggregation of nanoparticles [12]. Based on existing studies regarding the digestion of lipid nanoparticles, the ability of NLCs to control the digestion of the lipid matrix and the release of hydrophobic bioactive compounds during intestinal digestion remains limited [13,14,15].

Hydrogels are three-dimensional hydrophilic polymer networks formed via the crosslinking of polymers, which allow them to absorb large amounts of water while maintaining their structural integrity [16]. Hydrogels possess tunable physical properties and controllable degradability, enabling them to regulate the release of encapsulated bioactive compounds. Therefore, hydrogels are widely used as delivery systems in cosmetics, foods, and pharmaceuticals [16,17]. Polymer crosslinking for hydrogel formation occurs via two mechanisms, including chemical crosslinking and physical crosslinking [16]. The stability of physical crosslinking is generally lower than that of chemical crosslinking. However, under specific conditions (appropriate temperature, ionic strength, or pH), the strength of physical crosslinking can be sufficient to form stable hydrogels. Otherwise, the process of physical crosslinking is reversible, and environmental changes can induce the disintegration of the hydrogel structure. carrier systems based on hydrogels exhibit a variety of physical forms. By employing different preparation methods, hydrogels can be fabricated into diverse formats including blocks, coatings, films, and particles [18]. These distinct physical forms are designed to satisfy specific application requirements. Hydrogel beads are spherical three-dimensional colloidal systems [19]. Compared with large-volume block hydrogels, hydrogel beads have better dispersibility and are suitable to be added to the product as a semi-solid raw material carrying active ingredients, without changing the final physical state of the product. The product can maintain good fluidity by choosing an appropriate additional amount of hydrogel beads. The beads can be blended into beverages and dairy products (such as yogurt) without significantly altering the texture, as their size (micron-scale) and hydrogel-based structure mimic common food colloids (such as alginate pearls in bubble tea) [20,21]. Hydrogel beads possess a relatively high specific surface area and exhibit enhanced sensitivity to environmental stimuli. Alterations in the external environment are more prone to induce structural changes in the hydrogel matrix of beads, facilitating the release of active ingredients under specific conditions [22]. Hydrogel bead-based delivery systems have been extensively employed for curcumin delivery [23,24,25]. However, hydrogel systems lack essential components to facilitate mixed micelle formation, consequently impairing the intestinal absorption efficiency of curcumin.

The lipid nanocarrier-filled hydrogel delivery system, an emerging innovation derived from conventional hydrogel delivery systems, has garnered increasing attention in recent years. This system effectively addresses the stability of lipid nanocarriers and enables hydrogels to serve as a delivery system for hydrophobic active ingredients. Lipid nanocarriers, such as emulsions and lipid nanoparticles, suffer from flocculation and coalescence during storage due to Brownian motion. Immobilizing these carriers within the hydrophilic polymer network of hydrogels effectively restricts their mobility, thereby enhancing system stability [26]. Otherwise, the hydrogel network can restrict the diffusion of lipases within the hydrogel matrix and modulate the lipolysis rate of filled lipid nanocarriers [27]. Lipid nanocarrier-filled hydrogel delivery systems based on conventional emulsion-based delivery platforms (such as nanoemulsions) have been developed for curcumin delivery [28,29,30,31]. However, systems based on nanostructured lipid carriers, representing an advanced lipid nanocarrier system with multifaceted advantages, remain underexplored.

The main objective of this study was to develop NLC-filled hydrogel beads for the delivery of curcumin in foods. This system was designed to combine the advantages of NLCs (the protective ability for curcumin and the absorption-promoting performance) with those of hydrogel beads (high physical stability and controlled release performance in the intestinal tract). Alginate, an anionic polysaccharide, was used as the polymer to prepare hydrogel beads. In the presence of calcium cations, calcium alginate hydrogels are formed as a result of the formation of an egg-box structure [32]. The preparation process was studied and confirmed. The physicochemical characteristics of this delivery system were further investigated, as well as its protective effect on the stability of curcumin and antioxidant performance. Finally, the gastrointestinal fate of this delivery system was comprehensively analyzed through the combination of an in vitro digestion and ex vivo absorption model, with a focus on the intestinal absorption and antioxidant biological activity of the digestion products.

## 2. Materials and Methods

### 2.1. Materials

Curcumin was bought from Shaanxi Sciphar Natural Products Co., Ltd. (Xi’an, China). Sodium alginate was provided by Qingdao Bright Moon Seaweed Co., Ltd. (Qingdao, China). Polysorbate 80 (Tween 80) was obtained from Guangzhou Runhua Chemical Co., Ltd. (Guangzhou, China. Polyglyceryl-6 laurate (P6) was supplied by Nikko Chemicals Shanghai Co., Ltd. (Shanghai, China). GMS was supplied by Suzhou Nanohealth Co., Ltd. (Suzhou, China). Medium-chain triglyceride (MCT) was purchased from Britz Networks Sdn. Bhd. (Melaka, Malaysia). 1,1-Diphenyl-2-picrylhydrazyl (DPPH) and sodium cholate were purchased from TCI (Shanghai, China). Pancreatic lipase and gastric pepsin were obtained from Sigma-Aldrich (St. Louis, MO, USA). DMEM, Trypsin, and phosphate-buffered saline were obtained from Hyclone (Shanghai, China). Malondialdehyde (MDA), superoxide dismutase (SOD), and catalase (CAT) test kits were purchased from Nanjing Jiancheng (Nanjing, China). All other chemicals utilized were of analytical grade.

### 2.2. Fabrication of Curcumin-Loaded NLCs

Curcumin-loaded lipid nanoparticles were prepared using the hot high-pressure homogenization method [33]. Solid lipid (GMS, 6%, *w*/*w*), liquid lipid (MCT, 4%, *w*/*w*), emulsifier (Tween 80, 5%, *w*/*w*), and co-emulsifier (P6, 3%, *w*/*w*) were weighed and mixed at 80 °C. A certain amount of curcumin (0.2%, *w*/*w*) was added to the molten mixture and stirred at 80 °C until complete dissolution. Subsequently, purified water (81.8%, *w*/*w*) preheated to 80 °C was poured into the mixture under continuous stirring at 600 rpm, followed by 10 min of stirring. The coarse emulsion was then homogenized for 1 min at 10,000 rpm using a preheated high-shear emulsifier (FA25, FLUKO, Essen, Germany) to obtain the pre-emulsion. Immediately afterward, the pre-emulsion was homogenized through a preheated high-pressure homogenizer (AH100D, ATS, Shanghai, China) at 500 bar for four cycles, yielding a high-temperature nanoemulsion. The curcumin-loaded NLC dispersion was finally obtained after cooling the nanoemulsion to room temperature.

### 2.3. Fabrication of Curcumin-Loaded NLC-Hydrogel Beads

Curcumin-loaded NLC-hydrogel beads based on calcium alginate were prepared using the extrusion method combined with the ion crosslinking method [34]. Sodium alginate solutions with varying concentrations (0.5%, 1%, and 1.5, *w*/*v*) were prepared by dissolving a measured amount of sodium alginate in purified water under continuous stirring at 500 rpm for 24 h, followed by 1 h of standing to remove air bubbles. The curcumin-loaded NLC dispersion was then mixed with the sodium alginate stock solution at a 1:1 (*v*/*v*) ratio under stirring at 200 rpm for 5 min at room temperature, obtaining NLC-sodium alginate sols with different alginate concentrations. The sol was dripped into a calcium chloride solution via a single-channel microinjection pump (LSP01-1A, Longer, Baoding, China) to form hydrogel beads. The injection pump was set to a flow rate of 0.5 mL/min, with a 5 cm distance between the syringe needle tip and the calcium chloride solution. The formed hydrogel beads were further crosslinked in calcium chloride solution at room temperature for a specified duration. After crosslinking, the calcium chloride solution was collected using a pipette, followed by two washes with purified water to remove impurities. The wash solutions were also collected, and the curcumin-loaded NLC-hydrogel beads were finally harvested. The collected calcium chloride crosslinking solution and wash solutions were combined to determine the encapsulation efficiency. The effects of different concentrations of sodium alginate (0.5%, 1%, and 1.5%, *w*/*v*), calcium chloride concentrations (2.5%, 5%, and 10%, *w*/*v*), and the continuous crosslinking time (30 min, 60 min, and 120 min) on curcumin-loaded NLC-hydrogel beads were investigated to confirm the preparation process.

### 2.4. Determination of Encapsulation Efficiency

The encapsulation efficiency of curcumin-loaded NLCs (EE_NLC_) was determined using the ultrafiltration centrifugation method. Briefly, 400 μL of curcumin-loaded NLC dispersion was precisely pipetted into an ultrafiltration centrifugal tube (10 kDa, Millipore, Billerica, MA USA). After centrifugation at 10,000 rpm for 30 min, the ultrafiltrate from the lower chamber of the ultrafiltration tube was collected. The amounts of curcumin in the ultrafiltrate and NLC dispersion were quantified by UV spectrophotometry (a-1900PC, Puyuan, Shanghai, China) at 425 nm. The EE_NLC_ was calculated using the following formula:

(1) $${\mathrm{EE}}_{\mathrm{NLC}}\;(\%)=(1\;-\;\frac{W_{F}}{W_{T}})\times 100\%$$

where W_T_ is the total amount of curcumin in the curcumin-loaded NLC dispersion, and W_F_ is the amount of curcumin in the ultrafiltrate (free curcumin).

The encapsulation efficiency of curcumin-loaded NLC-hydrogel beads (EE_HB_) was calculated by determining the content of curcumin leaked from the hydrogel beads during the crosslinking process. After the preparation of the hydrogel beads, the content of curcumin in the mixed solution of the calcium chloride crosslinking solution and the wash solutions was determined. The EE_HB_ was calculated according to the following formula:

(2) $${\mathrm{EE}}_{\mathrm{HB}}\;(\%)=(1\;-\;\frac{W_{M}}{W_{T}})\;\times \;100\%$$

where W_T_ is the total amount of curcumin in the curcumin-loaded NLC dispersion, and W_M_ is the amount of curcumin in the mixed solution of the calcium chloride crosslinking solution and the wash solutions.

### 2.5. Determination of Particle Size

The particle size of the NLCs was measured using dynamic light scattering (ZS90, Malvern, Worcestershire, UK). Measurements were performed at a scattering angle of 90°, wavelength of 633 nm, temperature of 25 °C, and equilibration time of 2 min. Particle size distributions are reported as intensity distributions. Prior to analysis, samples were diluted to achieve an appropriate scattering intensity and loaded into polystyrene cuvettes for measurement.

### 2.6. Scanning Electron Microscope (SEM)

Freeze-dried curcumin-loaded NLC-hydrogel beads and blank alginate beads were mounted on a sample stage affixed with conductive adhesive tape and observed using scanning electron microscopy (Ultra Plus, Zeiss, Oberkochen, Germany).

### 2.7. X-Ray Diffraction (XRD)

Curcumin-loaded NLCs and curcumin-loaded NLC-hydrogel beads were characterized using X-ray diffraction (Smartlab, Rigaku, Tokyo, Japan). Before the measurement, the samples were subjected to freeze-drying treatment. The scanning parameters included an angular range of 5° to 40°, a scanning rate of 5°/min, and a step size of 0.02°.

### 2.8. Rheological Analysis

The rheological properties of samples were measured using a rheometer (MCRS40, Thermo Fisher, Waltham, MA, USA). All measurements were conducted at 25 °C. Analyses were performed over a shear rate range of 0.01 s^−1^ to 100 s^−1^, with the corresponding viscosity recorded.

### 2.9. Stability Study

Curcumin-loaded NLC-hydrogel beads prepared with different alginate concentrations, curcumin-loaded NLCs, and curcumin ethanol solutions were transferred to glass bottles and sealed with plastic lids. Then, the samples were exposed to ambient room temperature under natural light to evaluate the photostability. At predetermined intervals, the curcumin content was measured. In addition, the storage stability tests were conducted in darkness at temperatures of 25 °C and 40 °C. After 30 days of storage, the curcumin content was examined to assess stability.

### 2.10. DPPH Free Radical Scavenging Assay

Curcumin ethanol solution (0.2%, *w*/*v*), curcumin-loaded NLC-hydrogel beads, or curcumin-loaded NLCs (0.1 mL) were mixed with DPPH ethanol solution (3.9 mL). Subsequently, the mixed solution was kept in the dark at room temperature for 30 min. The absorbance of this solution at 517 nm was measured using a spectrophotometer. The DPPH radical scavenging activity was then calculated using the following formula:

(3) $$\mathrm{Scavenging}\;\mathrm{activity}\;(\%)=\frac{A_{C}-\;(A_{S}-A_{B})}{A_{C}}\;\times \;100\%$$

where A_S_ represents the absorbance of the mixture comprising 3.9 mL of DPPH stock solution and 0.1 mL of the test sample; A_C_ indicates the absorbance of the mixture comprising 3.9 mL of DPPH stock solution with 0.1 mL of distilled water; and A_B_ represents the absorbance of the mixture comprising 3.9 mL of ethanol and 0.1 mL of the sample.

### 2.11. Swelling and Dissolution Study

Enzyme-free simulated gastric fluid (SGF) and enzyme-free simulated intestinal fluid (SIF) were selected as the swelling media [35]. A fixed quantity of hydrogel beads (prepared by 6 mL of NLC-sodium alginate sol) was placed in 100 mL of the respective medium. The swelling medium was incubated at 37 °C for 120 min with agitation at 200 rpm. Hydrogel beads were retrieved from the dissolution medium at predetermined intervals, and the average diameter was measured. The swelling degree of the hydrogel beads was calculated using the following formula:

(4) $$\mathrm{Swelling}\;\mathrm{degree}\;\left(\%\right)=\frac{D_{S}\;-\;D_{B}}{D_{B}}\;\times \;100\%$$

where D_S_ is the average diameter of the hydrogel beads at the sampling point, and D_B_ is the initial average diameter of the hydrogel beads.

### 2.12. Dissolution Study

A fixed quantity of hydrogel beads (prepared by 10 mL of NLC-sodium alginate sol) was placed in 900 mL of the enzyme-free SGF. The dissolution medium was incubated at 37 °C for 120 min with agitation at 50 rpm. At predetermined intervals, a certain amount of the dissolution medium was collected to quantify the curcumin content, and an equivalent volume of pre-warmed medium was added back. After two hours of the experiment, the pH of the dissolution medium was adjusted to 6.8 through the rapid addition of NaOH solution, and the experiment was then extended for an additional 4 h.

### 2.13. In Vitro Simulated Digestion Study

The digestion behavior of curcumin-loaded NLCs and curcumin-loaded NLC-hydrogel beads was investigated using an in vitro two-step simulated digestion, according to the reported protocol with some modifications [36]. The impact of digestion on NLCs was evaluated by monitoring the changes in particle size. Lipid digestion kinetics were assessed by quantifying free fatty acid (FFA) release. Prior to the experiment, the original NLC dispersion was diluted 1:1 (*v*/*v*) with purified water to ensure comparability across samples.

Diluted curcumin-loaded NLCs and a quantified amount of NLC-hydrogel beads were separately mixed with 10 mL of simulated gastric fluid (pH 1.2, 3.2 mg/mL pepsin). The mixture was incubated at 37 °C under continuous agitation at 100 rpm for 2 h. After gastric digestion, the particle size of the nanoparticles in digestive fluid was measured.

The pH of the digesta from the above gastric stage was adjusted to 7.5 using NaOH and mixed with simulated intestinal fluid (pH 7.5, 4 mg/mL pancreatic lipase, 4.3 mg/mL sodium cholate, and 0.6 mM CaCl_2_). The mixed solution was maintained at 37 °C under 100 rpm agitation for 2 h. The FFA release during intestinal digestion was quantified using the pH-stat method, where 0.1 M NaOH was titrated to maintain the system pH at 7.5.

At various time points during intestinal digestion, a certain volume of digestive fluid was collected and centrifuged at 12,000 rpm for 15 min. The micellar phase was collected, and the curcumin content was quantified. Bioaccessibility was calculated using the following formula:

(5) $$\mathrm{Bioaccessibility}=(\frac{Q_{m}}{Q_{d}})\;\times \;100\%\;$$

where Q_m_ is the amount of curcumin in the micelle fraction, and Q_d_ is the amount of curcumin in the digestive fluid.

### 2.14. Ex Vivo Intestinal Permeation Assessment

The intestinal permeability of the digestion products was evaluated using a non-everted small intestinal sac method [37]. Male Sprague–Dawley rats (450–550 g; Shanghai SLAC Laboratory Animal Co., Ltd., Shanghai, China) were housed in the animal facility of Nantong University. All animal experimental protocols were approved by the Animal Ethics Committee of Nantong University. Intestinal digestion products of curcumin-loaded NLCs and curcumin-loaded NLC-hydrogel beads were collected from the in vitro simulated digestion (Section 2.11). Following 12 h of fasting, animals were anesthetized via intraperitoneal sodium pentobarbital injection. A midline laparotomy exposed the abdominal cavity, from which a 7 cm intestine segment was excised. The intestinal lumen was gently flushed with Krebs–Ringer buffer to eliminate chyme. One end of the intestine segment was ligated with surgical silk, followed by injection of the digestion products through a blunt syringe. The other end was subsequently secured to create a closed sac. Then, the prepared sacs were immersed in 15 mL of Krebs–Ringer buffer at 37 °C. After 120 min of incubation under dark conditions, the buffer solution and the excess samples within intestinal sacs were collected for analysis. P_app_ and the absorption ratio were calculated using the following equations.

(6) $$P_{\mathrm{app}}\;=\;\frac{\Delta Q}{A\;\times \;C_{0}\;\times \;\Delta t}$$

(7) $$\mathrm{Absorption}\;\mathrm{ratio}=(1\;-\;\frac{Q_{r}}{Q_{0}})\;\times \;100\%\;$$

where ΔQ/Δt indicates the quantity of curcumin transported across the intestinal sac over the time interval Δt, A represents the effective surface area of the intestinal sac, C_0_ indicates the concentration of curcumin inside the sac at the initial time point, Q_0_ represents the quantity of curcumin at the initial time point, and Qr indicates the residual amount of curcumin left in the intestinal sac.

### 2.15. Antioxidant Bioactivity Study

The antioxidant bioactivity of the digestion products from curcumin-loaded NLCs and curcumin-loaded NLC-hydrogel beads was evaluated according to a previously described procedure with some modifications [38,39]. The Caco-2 cells were grown in DMEM containing FBS (10%, *v*/*v*), penicillin–streptomycin (1%, *v*/*v*), and non-essential amino acids (1%, *v*/*v*) and cultured at 37 °C in an atmosphere of 5% CO_2_ and 95% relative humidity. Caco-2 cells with a cell density of about 1 × 10^5^ cells/mL were inoculated in 6-well plates and cultured in an incubator for 24 h. Then, the cells were treated with DMEM containing digestion fluid (diluted 100-fold) from NLCs and NLC-hydrogel beads. For the control group and the H_2_O_2_ group, the cells were cultured in DMEM. After 12 h of culture, the cells were exposed to H_2_O_2_ for 6 h, while the control group remained untreated. After washing with PBS buffer, the cells were lysed on ice and then centrifuged at 10,000 rpm for 20 min to obtain the supernatant. The levels of malondialdehyde (MDA), superoxide dismutase (SOD), and catalase (CAT) were subsequently measured using commercial kits in accordance with the manufacturer’s instructions.

### 2.16. Statistical Analysis

All experiments were performed in triplicate, with results presented as the mean ± standard deviation. Statistical analysis was carried out using Student’s *t*-test, and a *p* < 0.05 was considered a significant difference.

## 3. Results and Discussion

### 3.1. Encapsulation Efficiency of Curcumin-Loaded NLC-Hydrogel Beads

**Figure 1:**
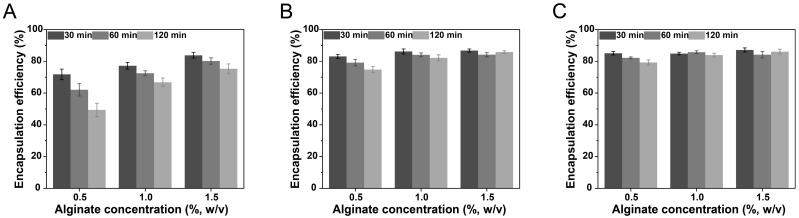
The influence of alginate concentration, crosslinking agent (calcium chloride) concentration, and crosslinking time on encapsulation efficiency of curcumin-loaded NLC-hydrogel beads. (**A**) 2.5% (*w*/*v*) calcium chloride concentration; (**B**) 5% (*w*/*v*) calcium chloride concentration; (**C**) 10% (*w*/*v*) calcium chloride concentration.

The encapsulation efficiency of curcumin in curcumin-loaded NLC-hydrogel beads was investigated to assess the preparation technology. The curcumin-loaded NLC dispersion system contained curcumin encapsulated in NLCs and unencapsulated curcumin (free curcumin in the aqueous phase), which might cause the rapid leakage of curcumin from hydrogel beads during the crosslinking process. Therefore, the encapsulation efficiency of curcumin-loaded NLCs was measured prior to the study of the preparation technology. The encapsulation efficiency of curcumin in freshly prepared NLCs was measured to be (91.57 ± 0.78)%, indicating that the prepared NLCs exhibited excellent encapsulation capability for curcumin. The influences of the alginate concentration, crosslinking agent (calcium chloride) concentration, and crosslinking time on the encapsulation efficiency were investigated. As illustrated in Figure 1, under all investigated conditions, the encapsulation efficiency of curcumin-loaded NLC-hydrogel beads was consistently lower compared to that of curcumin-loaded NLCs. This result suggested that, in addition to the inherent free curcumin in the aqueous phase, curcumin encapsulated in NLCs was also released from the hydrogel beads during the crosslinking process. As the crosslinking time increased, the curcumin-loaded NLCs progressively leaked into the crosslinking solution, resulting in a decrease in the encapsulation efficiency of NLC-hydrogel beads. The alginate concentration utilized for the preparation of NLC-hydrogel beads, as well as the calcium chloride concentration in the crosslinking solution, influenced the encapsulation efficiency of NLC-hydrogel beads. This is because both the polymer and crosslinking agent concentrations influenced the compactness of the hydrogel structure [40]. The increase in the polymer and crosslinking agent concentrations might inhibit the release of NLCs from hydrogel beads and thereby the leakage of encapsulated curcumin, leading to an increase in the encapsulation efficiency. When the concentration of calcium chloride was 10% and the concentration of alginate exceeded 1%, the encapsulation efficiency remained consistently high (above 80%) without any significant decrease even as the crosslinking time increased. These findings suggested that the hydrogel beads formed under these conditions could effectively encapsulate NLCs. For the optimized system (1% sodium alginate and 10% calcium chloride), the effective concentration of curcumin (drug loading content) could reach 0.858 ± 0.009 mg/g.

### 3.2. Physicochemical Characteristics

**Figure 2:**
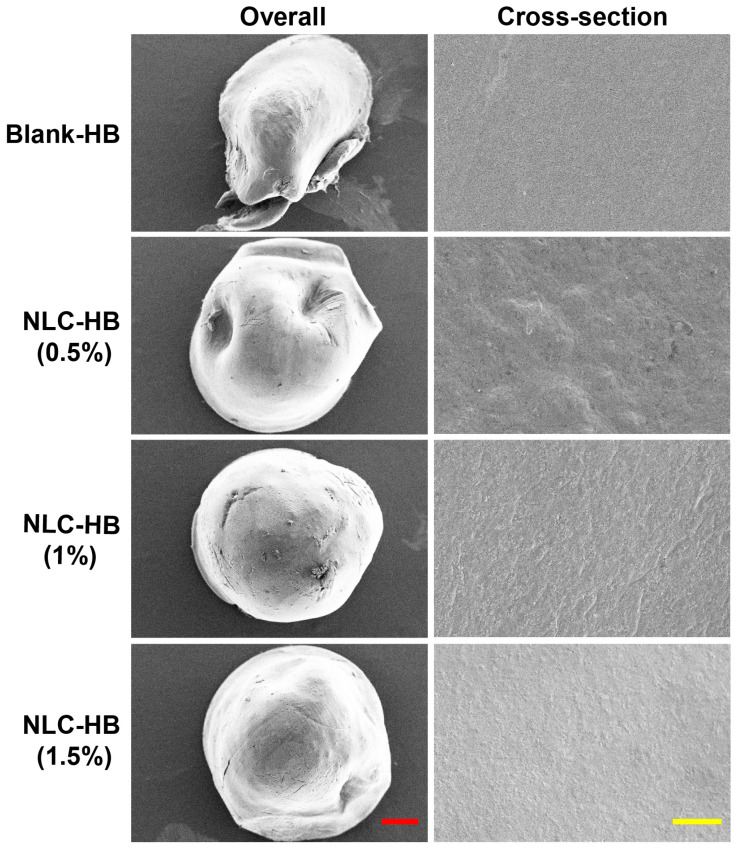
Overall and cross-section SEM images of blank hydrogel beads (0.5%) and curcumin-loaded NLC-hydrogel beads prepared with different alginate concentrations (red bar, 200 μm; yellow bar, 10 μm).

**Figure 3:**
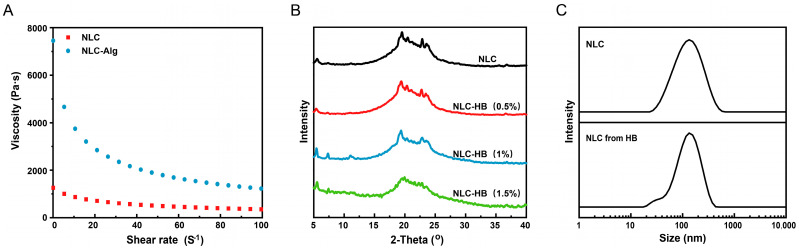
(**A**) Apparent viscosity of NLC-alginate mixture and alginate sol as a function of shear rate. (**B**) XRD diffractogram of curcumin-loaded NLCs and curcumin-loaded NLC-hydrogel beads prepared with varying alginate concentrations. (**C**) Particle size distributions of curcumin-loaded NLCs and nanoparticles released from curcumin-loaded NLC-hydrogel beads (0.5% alginate).

The overall and internal SEM images of blank hydrogel beads and curcumin-loaded NLC-hydrogel beads prepared with different alginate concentrations are displayed in Figure 2. The curcumin-loaded NLC-hydrogel beads maintained spherical or ellipsoidal shapes after the drying process. In contrast, the blank hydrogel beads displayed pronounced surface wrinkling and an irregular morphology. Blank hydrogel beads exhibited a smoother cross-sectional surface, whereas NLC-hydrogel beads displayed markedly rougher cross-sectional surfaces. Moreover, a reduction in the alginate concentration correlated with the progressively diminished roughness of the cross-sectional surface. Lipid nanoparticles, as insoluble solid particles, can function as fillers occupying the spaces within alginate gel networks, leading to an increase in the morphology roughness of filled hydrogel beads [41]. The filled hydrogel beads also demonstrated improved structural stability, effectively inhibiting shrinkage during the drying process. These results confirmed the successful incorporation of NLCs within the alginate hydrogel matrix. The rheological test (Figure 3A) demonstrated that the apparent viscosity of the alginate–NLC mixtures progressively decreased with increasing shear rates, indicative of shear-thinning behavior. Furthermore, the alginate–NLC mixtures exhibited significantly higher apparent viscosity and enhanced viscoelastic properties compared to pure sodium alginate sols. As illustrated in Figure 3B, the NLCs exhibited distinct diffraction peaks at approximately 19.7° and 23°, indicating that the solid lipid within the NLCs existed as a mixture of β- and β’-crystalline forms. Similarly, all NLC-hydrogel beads displayed prominent diffraction peaks at around 19.7° and 23°, consistent with the characteristic peaks observed in NLCs. This finding confirms that the lipid matrix within the hydrogel beads remained in a solid state, retaining the β- and β’-crystalline forms. Therefore, the fabrication of NLC-hydrogel beads had no significant influence on the structure of curcumin-loaded NLCs. After the preparation of NLC-hydrogel beads, the egg-box structure of the calcium alginate hydrogel was disrupted by immersing the beads in a citric acid solution. This process caused the hydrogel beads to disintegrate and release the encapsulated NLCs. The recovered NLCs were subsequently analyzed to evaluate the impact of the hydrogel matrix on the properties of the NLCs. The original NLCs had a mean particle size of 96.5 ± 3.2 nm, and the recovered NLCs exhibited a mean particle size of 99.2 ± 2.5 nm. There was no significant difference in the particle size distribution between the original NLCs and the recovered NLCs, indicating that the basic characteristics of the NLCs were preserved (Figure 3C).

### 3.3. Storage Stability 

**Figure 4:**
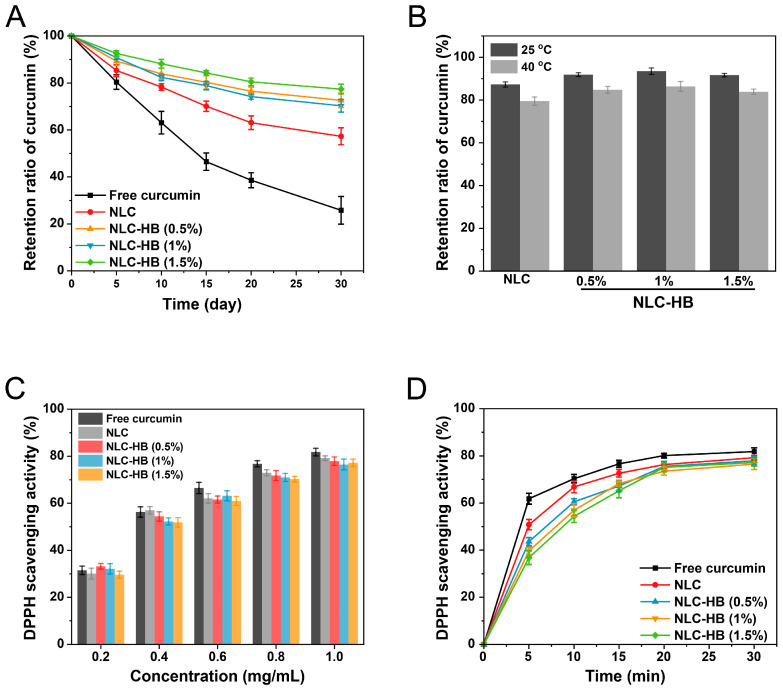
(**A**) Curcumin retention ratio of curcumin-loaded NLC-hydrogel beads prepared with varying alginate concentrations, curcumin-loaded NLCs, and curcumin ethanol solution (free curcumin) exposed to natural light at room temperature. (**B**) Curcumin retention ratio after 30 days of storage at 25 °C and 40 °C. (**C**) DPPH radical scavenging ability of samples at different concentrations. (**D**) Time-dependent DPPH radical scavenging ability of samples.

Light stability is an important factor affecting the application of curcumin. Lipid nanoparticles are considered to have the ability to improve the light stability of hydrophobic active ingredients [42]. The effect of hydrogel beads on the light stability of curcumin encapsulated within NLCs was investigated. The retention ratios of curcumin in ethanol solution (free curcumin), NLCs, and NLC-hydrogel beads exposed to natural light at room temperature are shown in Figure 4A. Under natural light conditions, the retention ratio of curcumin in all the samples exhibited a significant downward trend. After 30 days of storage, the retention ratio of curcumin in the ethanol solution decreased to 25.8%. After encapsulating curcumin in NLCs, the retention rate of curcumin increased to 57.3%. This is because the solid lipid matrix of the NLCs could reflect light. Further enhancement in curcumin stability was observed when NLCs were incorporated into hydrogel beads. At all alginate concentrations, the retention ratios of curcumin within NLC-hydrogel beads exceeded 70%. This might be due to the ability of hydrogels to scatter UV, which could weaken the irradiation of UV on curcumin-loaded NLCs and thereby increase the stability of encapsulated curcumin [43]. Furthermore, studies on the storage stability at various temperatures demonstrated that hydrogel beads significantly enhanced the storage stability of curcumin encapsulated in NLCs (Figure 4B). This finding further corroborated the protective efficacy of NLC-hydrogel beads on curcumin. Previous studies have also reported that hydrogel beads based on alginate could further improve the stability of curcumin encapsulated in nanocarriers [44].

### 3.4. In Vitro Antioxidant Study

The in vitro antioxidant ability of curcumin in samples was evaluated through the DPPH free radical scavenging assay. As there is an unpaired electron in the molecular structure, DPPH could be used as the free radical to assess the free radical scavenging capacity [45]. In the present study, the DPPH radical scavenging ability of curcumin-loaded NLCs and curcumin-loaded NLC-hydrogel beads was compared with curcumin ethanol solution (free curcumin). As shown in Figure 4C, the DPPH scavenging ability of all samples demonstrated an upward trend as the curcumin concentration was increased. Compared to free curcumin, the DPPH radical scavenging ability of curcumin-loaded NLCs and curcumin-loaded NLC-hydrogel beads showed a slight decrease. This decrease might be attributed to the encapsulation of curcumin by the carrier system, which impeded the diffusion of curcumin and consequently reduced the reaction rate. Additionally, the high temperatures during the preparation process might have caused the oxidation of curcumin, thereby diminishing the radical scavenging capacity. Further investigation was conducted on the time-dependent DPPH radical scavenging capacity of curcumin-loaded NLC-hydrogel beads (Figure 4D). The result revealed that compared to free curcumin, both curcumin-loaded NLCs and curcumin-loaded NLC-hydrogel beads exhibited reduced DPPH radical scavenging efficiency. These findings further confirmed that the solid lipid matrix of the NLCs might influence the radical scavenging capacity by influencing the diffusion of curcumin.

### 3.5. Swelling and Dissolution Characteristics

**Figure 5:**
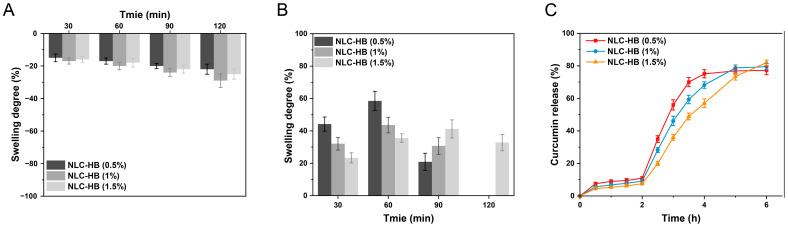
(**A**) The swelling degree of curcumin-loaded NLC-hydrogel beads with varying alginate concentrations in enzyme-free SGF. (**B**) The swelling degree of curcumin-loaded NLC-hydrogel beads with varying alginate concentrations in enzyme-free SIF. (**C**) Dissolution profiles of curcumin-loaded NLC-hydrogel beads with varying alginate concentrations (0–2 h: pH 1.2; 2–6 h: pH 6.8).

Hydrogel beads based on calcium alginate exhibit significant swelling and disintegration in gastrointestinal fluid, which is crucial in controlling digestion and release. Therefore, the swelling behavior of NLC-hydrogel beads in enzyme-free SGF and SIF was studied. As illustrated in Figure 5A, the hydrogel beads exhibited pronounced contraction in SGF. This phenomenon is derived from the weakened electrostatic repulsion between the alginic acid molecular chains under acidic conditions, which promoted chain coiling and aggregation, thereby inducing macroscopic shrinkage. As shown in Figure 5B, the swelling degree of the NLC-hydrogel beads in SIF was related to the concentration of alginate used in the preparation process. Within the first 60 min, the reduction in the alginate concentration increased the swelling degree. This phenomenon could be attributed to the presence of phosphates and monovalent cations in SIF, which facilitated the dissociation of calcium ions from the egg-box structure of the calcium alginate hydrogel, leading to the swelling of the beads. Hydrogel beads prepared with a higher alginate concentration possessed a more compact structure, thereby delaying the swelling. Furthermore, after 60 min, the swelling degree exhibited a downward trend. This was related to the erosion and dissociation of the hydrogel matrix, which resulted in a reduction in the size of the hydrogel beads. At lower sodium alginate concentrations (0.5% and 1%), this erosion and dissociation caused the hydrogel beads to disintegrate.

As illustrated in Figure 5C, the release of curcumin from NLC-hydrogel beads in media of pH 1.2 was relatively low. This limited release could be attributed to the pore size of the hydrogel beads being smaller than the mean size of the NLCs. The curcumin released from the hydrogel beads was mainly in free form, which was not influenced by the polymer concentration. Furthermore, a minor proportion of smaller nanoparticles might also diffuse out from the hydrogel beads. This diffusion process was influenced by the alginate hydrogel matrix, resulting in a relatively higher release rate under a low alginate concentration. Curcumin encapsulated in NLC-hydrogel beads exhibited a higher release rate in media of pH 6.8 compared to pH 1.2. Higher alginate concentrations reduced the cumulative release rate of curcumin between 2 and 4 h, accompanied by a more gradual release profile. This phenomenon can be attributed to the fact that the increasing concentration of alginate delayed the swelling process of the hydrogel beads. As swelling occurred, the pore size of the hydrogel beads increased, facilitating the gradual diffusion of the NLC out of the hydrogel matrix and consequently promoting the release of curcumin. However, this swelling-dependent diffusion process was inhibited by the increase in the alginate concentration.

### 3.6. In Vitro Digestion Study

**Figure 6:**
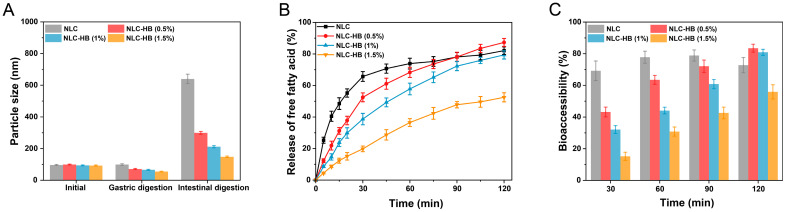
(**A**) The mean size of NLCs before digestion and after each digestion stage, and NLCs released from NLC-hydrogel beads (with varying alginate concentrations) into digestive fluid after each digestion stage. (**B**) Release profiles of free fatty acids from curcumin-loaded NLCs and curcumin-loaded NLC-hydrogel beads with varying alginate concentrations during intestinal digestion. (**C**) The variation of curcumin bioaccessibility during intestinal digestion.

Figure 6A displays the mean size of the nanoparticles in digestive fluid after simulated digestion. After gastric digestion, the mean particle size of the original NLCs remained unchanged, indicating that gastric digestion had no significant effect on the structural integrity of NLCs. For NLC-hydrogel beads, the size of the nanoparticles in digestive fluid after gastric digestion was smaller compared to the initial size. The measured reduction in mean particle size could be attributed to the diffusion of only smaller particles from the hydrogel beads into the digestive fluid during gastric digestion. For the original NLCs, the particle size in the digestive fluid increased to above 600 nm after intestinal digestion, indicating significant particle aggregation. However, for the NLC-hydrogel beads, the size of the nanoparticles in the digestive fluid was relatively smaller. Notably, when the alginate concentration was 1.5%, the size of the nanoparticles in the digestive fluid was measured to be only 173 nm after intestinal digestion. This demonstrated that the hydrogel beads could effectively inhibit the aggregation of NLCs during intestinal digestion. This phenomenon was attributed to the gradual release of nanoparticles from the hydrogel beads into the digestive fluid, thereby maintaining the relatively low nanoparticle concentrations and consequently reducing the probability of collision-induced aggregation.

In this study, the pH-stat method was used to determine the release of free fatty acids during intestinal digestion. The release curves of free fatty acids from the original NLCs and NLC-hydrogel beads are shown in Figure 6B. For NLCs, the release rate of free fatty acids was relatively fast at the initial stage, and then the release rate gradually decreased. Under all alginate concentrations, NLC-hydrogel beads showed a slower release rate of free fatty acids at the initial stage, with a more moderate lipolysis process. This might be due to the fact that the hydrogel beads affect the contact between the lipase and the nanoparticles, which was the requirement to initiate the lipolysis reaction [46]. There might be two different pathways for the lipid digestion of NLC-hydrogel beads: (1) NLCs diffused from the hydrogel beads into the digestive fluid and were subsequently hydrolyzed by lipase; and (2) lipase diffused into the hydrogel beads and participated in the digestion of NLCs within the beads. Both of these pathways were influenced by the hydrogel matrix. Firstly, NLCs were gradually released from the hydrogel beads into the digestive fluid, where they underwent sequential lipolysis by lipase. Additionally, the hydrogel matrix restricted lipase diffusion into the beads, leading to a relatively slow digestion rate of NLCs within the beads [47]. With the increase in the alginate concentration, both the rate and extent of lipolysis exhibited a downward trend. This is because the increase in the alginate concentration concurrently inhibited both digestion pathways. Due to the tightly crosslinked structure, NLC-hydrogel beads prepared with higher alginate concentrations could effectively prevent lipase from diffusing into the hydrogel beads and simultaneously hinder the diffusion of NLCs within the beads into the digestive fluid. It is noteworthy that at the 0.5% alginate concentration, the release amount of free fatty acids from NLC-hydrogel beads after intestinal digestion exceeded that of the original NLC group. This phenomenon might be attributed to the hydrogel beads preventing the aggregation of lipid nanoparticles during digestion, thereby increasing the surface area available for lipase adsorption and facilitating digestion [48].

The fatty acids generated from lipolysis could form mixed micelles together with bile salts and surfactants during intestinal digestion. Hydrophobic active components could be encapsulated within these mixed micelles, facilitating their absorption in the small intestine [49]. Bioaccessibility, defined as the percentage of solubilized hydrophobic active compounds in the gastrointestinal tract, is related to the micellization degree of hydrophobic active components during digestion [50]. The changes in the bioaccessibility of curcumin during the intestinal digestion of curcumin-loaded NLCs and NLC-hydrogel beads are shown in Figure 6C. For NLCs, the bioaccessibility of curcumin exceeded 65% within the first 30 min of intestinal digestion. As digestion progressed, there was no significant change in the curcumin bioaccessibility. This result indicated a limited capacity of the prepared NLCs to control the release of curcumin during intestinal digestion. Similar to the effect observed in lipolysis, the alginate concentration significantly influenced the bioaccessibility of curcumin. As the alginate concentration increased, the bioaccessibility of curcumin at the early stage of intestinal digestion exhibited a downward trend. This phenomenon was attributed to the fact that the bioaccessibility of hydrophobic active components depended on the lipolysis extent of the lipid matrix. The undigested lipid matrix of NLCs impeded the release of curcumin into mixed micelles. Otherwise, the formation of digestion products, such as free fatty acids and mixed micelles, was decreased by the suppressed lipolysis process. Previous studies have reported that the degree of lipolysis had a significant influence on the bioaccessibility of hydrophobic active components [14,51]. Compared with NLCs, NLC-hydrogel beads exhibited a sustained-release profile. After 30 min of intestinal digestion, the bioaccessibility of curcumin in NLC-hydrogel beads was lower than that in NLCs. As digestion progressed, the bioaccessibility gradually increased. These results were related to the influence of the hydrogel beads on lipid digestion. Hydrogel beads could control the digestion rate of NLCs within the beads by restricting lipase and NLC diffusion, thereby regulating the formation of mixed micelles and the release rate of curcumin into micelles.

### 3.7. Ex Vivo Permeation and Absorption of Curcumin

**Figure 7:**
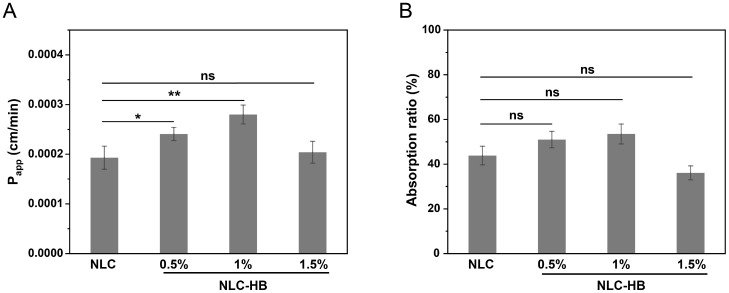
(**A**) The apparent permeability (P_app_) of curcumin in digestion products from curcumin-loaded NLCs and curcumin-loaded NLC-hydrogel beads with varying alginate concentrations. (**B**) The absorption ratio of curcumin in digestion products from curcumin-loaded NLCs and curcumin-loaded NLC-hydrogel beads with varying alginate concentrations. ** *p* < 0.01, * *p* < 0.05, ns indicates no significance.

Curcumin exhibits limited absorption and bioavailability, primarily due to its poor water solubility and susceptibility to degradation in the intestinal environment. The permeability and absorption of curcumin from digestion products of NLCs and NLC-hydrogel beads were further investigated by combining in vitro digestion with ex vivo permeability. The ex vivo permeability model based on a small intestinal sac possessed complete physiological functions of the intestine and was capable of mimicking the intricate transmembrane transport and absorption processes [52]. The apparent permeability (P_app_) and absorption ratio of curcumin from the digestion products are demonstrated in Figure 7. The influence of the NLC-hydrogel beads on the permeability of curcumin in NLCs after digestion was related to the concentration of alginate used for preparing the NLC-hydrogel beads. There was no significant difference in the absorption ratio of curcumin from the digestion products of NLCs and NLC-hydrogel beads. Compared to NLCs, the P_app_ of curcumin from the digestion products of NLC-hydrogel beads (0.5% and 1%) significantly increased. However, when the alginate concentration was raised to 1.5%, there was no significant difference in the P_app_ between NLC-hydrogel beads and NLCs. The promoting permeation effect of hydrogel beads might be mainly related to two factors. Firstly, as shown in the digestion study, curcumin encapsulated in NLC-hydrogel beads was gradually released from the lipid matrix and solubilized in the intestinal fluid, which was beneficial to the stability of curcumin. It has been reported that the encapsulation of curcumin in the carrier system could improve the intestinal absorption of curcumin by inhibiting the degradation of curcumin in gastrointestinal fluids [53,54]. In addition, alginate might increase the permeability of curcumin by increasing the electronegativity of the mixed micelles after digestion [55]. Under the 1.5% alginate concentration, the lower permeability might result from a larger portion of curcumin remaining encapsulated within the lipid matrix of NLC-hydrogel beads, thereby decreasing the intestinal permeation of curcumin. The above results indicated that by selecting an appropriate preparation process for hydrogel beads, NLC-hydrogel beads could significantly promote the intestinal permeation of curcumin.

### 3.8. Antioxidant Bioactivity of Digestion Products

**Figure 8:**
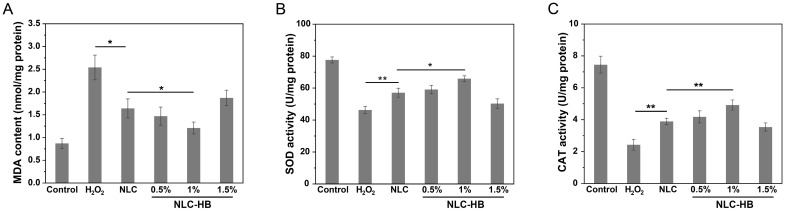
MDA content (**A**), SOD activity (**B**), and CAT activity (**C**) in H_2_O_2_-treated Caco-2 cells after treatment with digestion products from curcumin-loaded NLCs or curcumin-loaded NLC-hydrogel beads prepared with varying alginate concentrations. ** *p* < 0.01, * *p* < 0.05.

The antioxidant bioactivity of the digestion products from curcumin-loaded NLC-hydrogel beads was investigated by evaluating the levels of MDA, SOD, and CAT in Caco-2 cells exposed to the digestion products and hydrogen peroxide. MDA is a byproduct of lipid peroxidation induced by free radicals and can react with intracellular nucleic acids, proteins, and other biological macromolecules [56]. As illustrated in Figure 8A, compared to the control group, the MDA content in Caco-2 cells treated with hydrogen peroxide was significantly increased. After treatment with the digestion products of curcumin-loaded NLCs or NLC-hydrogel beads, the MDA contents in hydrogen peroxide-treated Caco-2 cells were decreased markedly, indicating that these digestion products containing curcumin could alleviate lipid peroxidation. Notably, the digestion products of curcumin-loaded NLC-hydrogel beads (1%) demonstrated a more pronounced reduction in the MDA content compared to the digestion products of curcumin-loaded NLCs. This effect might be attributed to the fact that the NLC-hydrogel beads with a suitable alginate concentration could protect curcumin from digestive fluid degradation during intestinal digestion, thereby enhancing the antioxidant bioactivity of the digestion products. Previous studies about the antioxidant activity of curcumin under gastrointestinal digestion have reported that the digestion products of encapsulated curcumin exhibited superior inhibitory effects on lipid peroxidation inside cells compared to those of non-encapsulated curcumin [38].

SOD and CAT are crucial antioxidant enzymes that play a vital role in the cellular defense against oxidative stress. SOD catalyzes the conversion of superoxide radicals into hydrogen peroxide, which is subsequently reduced by CAT. Compared with the control group, the exposure of Caco-2 cells to hydrogen peroxide resulted in a significant decrease in SOD and CAT activities (Figure 8B,C). However, treatment with the digestion products of curcumin-loaded NLCs or NLC-hydrogel beads led to a significant increase in SOD and CAT activities in hydrogen peroxide-treated Caco-2 cells. Moreover, the digestion products of NLC-hydrogel beads (1%) exhibited significantly higher SOD and CAT activities than those of NLCs. These findings were consistent with the MDA result, indicating that NLC-hydrogel beads prepared with a suitable alginate concentration could significantly enhance the antioxidant bioactivity of digestion products and effectively alleviate cellular oxidative damage. In addition to the effect on the antioxidant bioactivity of curcumin, alginate itself might contribute to oxidative stress mitigation, thereby promoting the overall antioxidant bioactivity of digestion products [57,58]. However, NLC-hydrogel beads with a high alginate concentration (1.5%) resulted in lower SOD and CAT activities compared to NLCs, possibly due to the restricted curcumin release from the delivery system.

## 4. Conclusions

In this study, NLC-filled hydrogel beads based on calcium alginate were developed for the delivery of curcumin. Various preparation parameters were evaluated to obtain a high encapsulation efficiency. The results of the physicochemical characteristics confirmed the feasibility of the preparation method and the successful incorporation of NLCs within the alginate hydrogel beads. The stability study indicated that NLC-filled hydrogel beads could further improve the stability of curcumin encapsulated in NLCs. The in vitro digestion research demonstrated that hydrogel beads adjusted the digestion characteristics of NLCs by controlling the contact between the lipase and NLCs, including inhibiting the aggregation of particles, controlling the lipolysis rate of the lipid matrix, and regulating the release rate of curcumin during intestinal digestion. The combination of an in vitro digestion with an ex vivo permeability study indicated that NLC-hydrogel beads prepared with appropriate alginate concentrations (0.5% and 1%) significantly promoted the intestinal permeation of curcumin encapsulated in NLCs. The antioxidant bioactivity study suggested that the digestion products of curcumin-loaded NLC-hydrogel beads (1%) demonstrated a more enhanced antioxidant bioactivity compared to those of curcumin-loaded NLCs. Overall, the NLC-hydrogel beads system was a suitable carrier system for the delivery of curcumin for food applications. These results offer valuable insights into the rational formulation design of NLC-hydrogel beads.

## Data Availability

All relevant data are included in the manuscript.
